# Time course measurements of leaf elevation angles during shade avoidance response in *Arabidopsis thaliana* using *Raspberry Pi* computers and computer vision technique

**DOI:** 10.17912/micropub.biology.001633

**Published:** 2025-07-28

**Authors:** ByungHoon B. Kim

**Affiliations:** 1 Department of Biology, University of North Alabama, Florence, Alabama, United States

## Abstract

Shade avoidance response in plants includes a higher leaf elevation angle. A cost-effective and noninvasive high throughput image analysis technique was used to measure the dynamics of leaf elevation angles during shade avoidance response in *Arabidopsis* . Time-lapse images were taken from the top and the side of a plant using *Raspberry Pi* computers. The leaf elevation index for each plant is determined from the plant dimensions measured by an image analysis software package *PlantCV* . This method was used to monitor the dynamics of changing leaf elevation angles in wild-type plants and in shade avoidance mutants *pif4-2pif5-3* and *pif7-2* plants.

**Figure 1. The concept of image analysis and the time course data:**
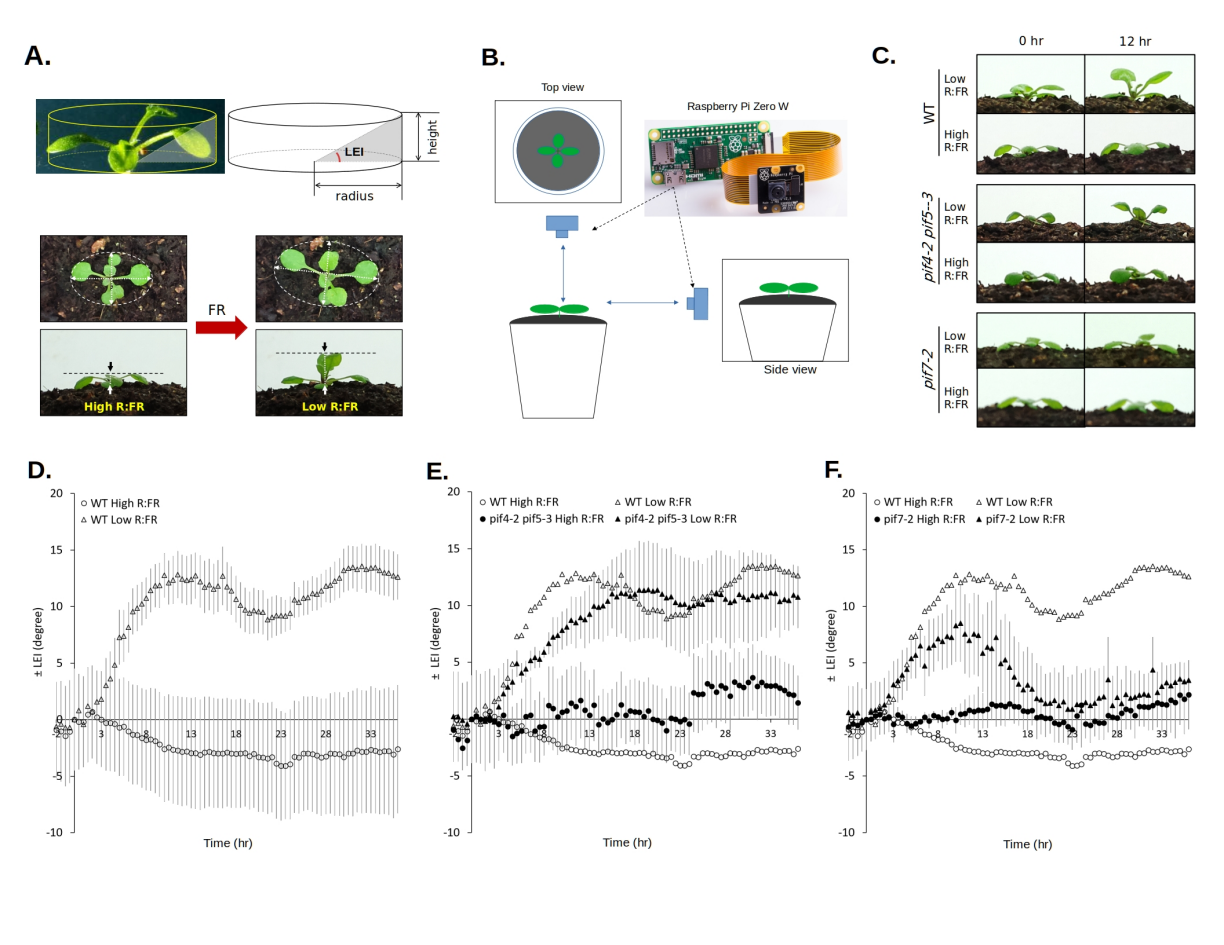
**(A)** A hypothetical concept that assumes a plant as a cylinder. Ellipse major axis, ellipse minor axis, and height are shown with dashed line arrows. Lowering the R:FR ratio by far-red light supplementation (FR) increased the height of the plant. **(B)** At a given time point, a top view and a side view pictures were taken by two separate *Raspberry Pi Zero W* units. **(C)** The images of representative plants at the onset of the low R:FR condition (0 hr) and after 12 hr from the treatment begin. **(D)(E)(F)** Changes in LEI over a time course (36 hr). Wild-type plants only (D). The results from *pif4-2pif5-3* mutants (E) and from *pif7-2* mutants (F) are superimposed with the results from wild-type plants shown in panel D. Error bars indicate standard error. WT high R:FR (n=8), WT low R:FR (n=9), *pif4-2pif5-3* high R:FR (n=3), *pif4-2pif5-3* low R:FR (n=3), *pif7-2* high R:FR (n=3), *pif7-2* low R:FR (n=3).

## Description

Light quality is one of the most important regulators of plant development. In a densely populated area such as a crop field, the red spectrum (R: λ = 660 nm) in the sunlight is selectively consumed by actively photosynthesizing plants while the far-red spectrum (FR: λ = 730 nm) is not consumed. As a result, a lower ratio between the red light and the far-red light (R:FR) is created in the surrounding area, which serves as a signal for impending competition with neighboring plants. The low R:FR conditions sensed by the plant trigger a series of developmental processes called shade avoidance response (elongational growth, higher leaf elevation angles, etc.) to better receive sunlight (Roig-Villanova and Martínez-García, 2016). The plant sensor for R:FR ratio is phytochrome, which is activated by red light and inactivated by far-red light. Under high R:FR conditions active phytochromes interact with transcription factors PIFs (PHYTOCHROME-INTERACTING FACTORs; *PIF1* , AT2G20180 ; *PIF3* , AT1G09530 ; *PIF4* , At2g43010 ; *PIF5* , At3g59060 ; *PIF7* , At5g61270 ) to inhibit their activity, while low R:FR conditions inactivate phytochromes, allowing the PIFs to be active (Lorrain et al., 2008). The PIF transcription factors in turn activate the transcription of various downstream regulators of shade avoidance response. Indeed, *phyB* (AT2G18790) mutants exhibit hyper-elongated phenotypes whereas various *pif* mutants exhibit a reduced shade avoidance response (Devlin et al., 1996; Li et al., 2012; Lorrain et al., 2008).

Due to the visible changes during the shade avoidance response, the phenotypes can be scored easily by measuring the physical parameters of plants such as hypocotyl length and chlorophyll content. However, conventional methods to measure phenotypes are often invasive and can only be done once because they terminate the plants. They also have limitations for measuring leaf elevation angles. These problems can be overcome by utilizing a computer vision technique that allows researchers to collect and analyze image data multiple times during the plants' lifetime. So far, various phenotype analysis systems have been developed. One such approach to measure leaf elevation angle uses expensive imaging tools including a robotic system (Michaud et al., 2023). Another system uses a simple and low-cost platform consisting of a Raspberry Pi computer and a camera (Oskam et al., 2024). Although the latter system is cost-effective and provides a high spatial resolution in measuring leaf elevation angles, it requires a target leaf to be arranged parallel to the camera before the images are taken. In addition, presence of another leaf between the target leaf and the camera limits the visibility of the target leaf and the feasibility of this system for measuring the leaf elevation angles unless the leaf blocking the target leaf is removed, which may introduce wounding stress to the plant.

Here, I introduce another low-cost image analysis solution for tracking the dynamics of leaf elevation status over a time course. This system does not have the above-mentioned limitations for the target leaf position and can be used to track a plant over a longer period of time. The main purpose of this approach was to develop a simple low-resolution tool for labs with budget constraints or for undergraduate research.

This approach also introduces a new simple concept of *leaf elevation index* ( *LEI* ) as an assay. Instead of measuring an actual angle of a single leaf, it uses overall plant architecture to assign a hypothetical angle (leaf elevation index) that represents the leaf elevation status of the plant ( Figure 1A ). This is based on the assumption that the shape of a plant with a rosette, such as Arabidopsis, is cylindrical. When the actual leaf elevation angle increases, the height of the plant (cylinder) increases. The leaf elevation index can be calculated from the measured radius (r) and height (h) of the cylinder (LEI = tan ^-1^ (h/r) x 180/π). Since the rosette of an Arabidopsis plant at this stage is not completely round, the mean value of the ellipse major axis and ellipse minor axis was used as a hypothetical diameter. The radius was determined from this diameter. Even though this approach does not measure the actual elevation angle of a particular leaf, this simple approach assigns a particular value (LEI) to a plant. This is useful in estimating the leaf elevation status of a whole plant and in assessing the shade avoidance response at this stage of plants (see below). To take time-lapse images from the top and from the side of a plant, two units of *Raspberry Pi Zero W* were used per plant ( Figure 1B ). The digital images were analyzed to measure the ellipse major axis, ellipse minor axis, and the height using an open-source software package *PlantCV* (Berry et al., 2018). Such a single image analysis method is automatically repeated for all images taken during the time course experiment.

This approach was successfully used to monitor the dynamics of leaf elevation status over a time course. The shade avoidance response in wild-type Arabidopsis plants was clearly detected with an increased *leaf elevation index* (LEI; Fig. 1 C D). Under low R:FR conditions the LEI increased to its maximum after about 10-hour treatment, while high R:FR conditions did not elicit this response. Although a continuous light condition was used in this experiment, a rhythmic fluctuation of LEI was also detected as shown in a previous report that used light/dark cycling environments (Michaud et al., 2023). In *pif4-2pif5-3* double mutant plants this response was slower and did not reach its maximum before 15 hr of treatment, which was also less pronounced when compared with wild-type plants ( Fig. 1 C E). In *pif7-2* mutant plants the response rate was similar to wild-type plants, but the maximum LEI was significantly lower. The leaf elevation under this condition was rather temporary, and the LEI came back down to its basal level after 20 hours of treatment ( Fig. 1 C F). These results are in line with the previous findings that *pif4-2pif5-3* and *pif7-2* mutants exhibit reduced hypocotyl length under low R:FR conditions (Li et al., 2012; Lorrain et al., 2008). Taken together, these results indicate the potential of this simple and cost-effective approach for low-resolution monitoring of leaf elevation status over a time course.

## Methods

**Plant growth and experimental conditions**

*Arabidopsis thaliana* ecotype Columbia wild-type and mutant ( *pif4-2pif5-3* and *pif7-2* ) seeds were surface sterilized with 30% Clorox ^®^ with 0.01% Triton X-100 for 10 min and rinsed with autoclaved water four times. Seeds were placed on Murashige-Skoog medium with 0.8% agar and cold-treated in a refrigerator for a week. Seedlings were grown at 23°C for a week under continuous light (80 µmol/m ^2^ /s; R:FR = 3). A single seedling was transferred to the center of a pot with soil and further grown for another week under the same condition. Then, the pots were placed in a growth chamber (continuous light; 120 µmol/m ^2^ /s; R:FR=2.5) installed with *Raspberry Pi* units (see below). The soil is kept moist by placing the pot in a tall Petri dish filled with water. After 24 hr of acclimation period for the environmental change, plants were treated with or without supplemental far-red light (final R:FR = 0.36 or 2.5).

**Imaging system and time-lapse imaging**

The imaging station was developed by modifying the previously reported system (Tovar et al., 2018). Each imaging station was composed of two *Raspberry Pi Zero W* units, one for top view and another for side view images ( Fig. 1B ). Each *Raspberry Pi* unit for side view images was placed on the wall of the growth chamber (18 cm away from each target plant) at the same height as the plants on the soil. A *Raspberry Pi* unit for top view images was placed on the ceiling of the growth chamber (18 cm directly above each target plant). All *Raspberry Pi* units were programed to take a picture every 30 min using *crontab* function in Linux Operating System. Images saved on all *Raspberry Pi* *Zero W* units were regularly transferred to a master unit ( *Raspberry Pi 4* ) connected to the same WiFi network ( *rsync* function in Linux OS).

**Image analysis**

Time-lapse images were analyzed using an open-source software package *PlantCV* (Berry et al., 2018). The entire procedure is well described in *Documentation for PlantCV* ( https://plantcv.readthedocs.io/en/stable/ ). The specific python script used in this study was from the above *Documentation* and is included as an extended data file ( *example_workflow.py* ). The top view images were used to obtain ellipse major axis and ellipse minor axis of rosettes, and the side view images were used to obtain height (elevation) of plants. Before an automated analysis of all time-lapse images for a particular plant, various parameters in this script ( *threshold* , *max_value* , *size,* *roi* , and *line_position* ) were adjusted by testing the script for a single exemplary image using *Jupyter Notebooks* (described in *Documentation* mentioned above). This step was repeated for several other images selected from the same time course experiment to ensure optimum image capture throughout the time course. Then, this adjusted script was automatically repeated for all images collected throughout the time course (see *workflow parallelization* in *Documentation* mentioned above). The resulting output file (json format) was converted to csv file for further analysis (see *PlantCV Utilities* section under *Accessory Tools* in *Documentation* mentioned above). Using the *ImageJ* program, the conversion factors between the values in pixels and the actual length in millimeter (mm) were determined by comparing the values with the length standard included in the images. Further analyses were carried out using Microsoft Excel. The mean value of the ellipse major axis and ellipse minor axis was used as the diameter of the rosette, and the radius was determined from this diameter. The LEI was determined from the radius (r) and height (h) of the hypothetical cylinder (LEI = tan ^-1^ (h/r) x 180/π) ( Fig. 1 A). Mean values and standard errors of three to nine plants' data were calculated. In each data set for a particular treatment, the changes in mean LEI values were normalized so that the value at the beginning of the treatment is set to zero (+/- LEI=0 at 0 min of treatment).

## Reagents

*Arabidopsis thaliana mutant plants used in this study*

**Table:** 

Ecotype	Genotype	Available From
Columbia	Wild type	
Columbia	*pif4-2pif5-3*	ABRC # CS68096
Columbia	*pif7-2*	ABRC # CS71656

## Data Availability

Description: python script used in this study. Resource Type: Software. DOI: https://doi.org/10.22002/q71sw-5vz65
